# The Genetic Aspects of Periodontitis Pathogenesis and the Regenerative Properties of Stem Cells

**DOI:** 10.3390/cells13020117

**Published:** 2024-01-09

**Authors:** Klaudia Ustianowska, Łukasz Ustianowski, Estera Bakinowska, Kajetan Kiełbowski, Joanna Szostak, Martyna Murawka, Bartosz Szostak, Andrzej Pawlik

**Affiliations:** 1 Department of Physiology, Pomeranian Medical University, 70-111 Szczecin, Poland; klaudusia527@wp.pl (K.U.); lukasz011235@wp.pl (Ł.U.); ebakinowska@op.pl (E.B.); kajetan.kielbowski@onet.pl (K.K.); martyna.murawka@pum.edu.pl (M.M.); bartosz.szostak@pum.edu.pl (B.S.); 2 Department of Experimental and Clinical Pharmacology, Pomeranian Medical University, 70-111 Szczecin, Poland; joannaszostak99@gmail.com

**Keywords:** periodontitis, gene polymorphism, epigenetics, stem cells, extracellular vesicles

## Abstract

Periodontitis (PD) is a prevalent and chronic inflammatory disease with a complex pathogenesis, and it is associated with the presence of specific pathogens, such as *Porphyromonas gingivalis*. Dysbiosis and dysregulated immune responses ultimately lead to chronic inflammation as well as tooth and alveolar bone loss. Multiple studies have demonstrated that genetic polymorphisms may increase the susceptibility to PD. Furthermore, gene expression is modulated by various epigenetic mechanisms, such as DNA methylation, histone modifications, or the activity of non-coding RNA. These processes can also be induced by PD-associated pathogens. In this review, we try to summarize the genetic processes that are implicated in the pathogenesis of PD. Furthermore, we discuss the use of these mechanisms in diagnosis and therapeutic purposes. Importantly, novel treatment methods that could promote tissue regeneration are greatly needed in PD. In this paper, we also demonstrate current evidence on the potential use of stem cells and extracellular vesicles to stimulate tissue regeneration and suppress inflammation. The understanding of the molecular mechanisms involved in the pathogenesis of PD, as well as the impact of PD-associated bacteria and stem cells in these processes, may enhance future research and ultimately improve long-term treatment outcomes.

## 1. Introduction

**Figure 1:**
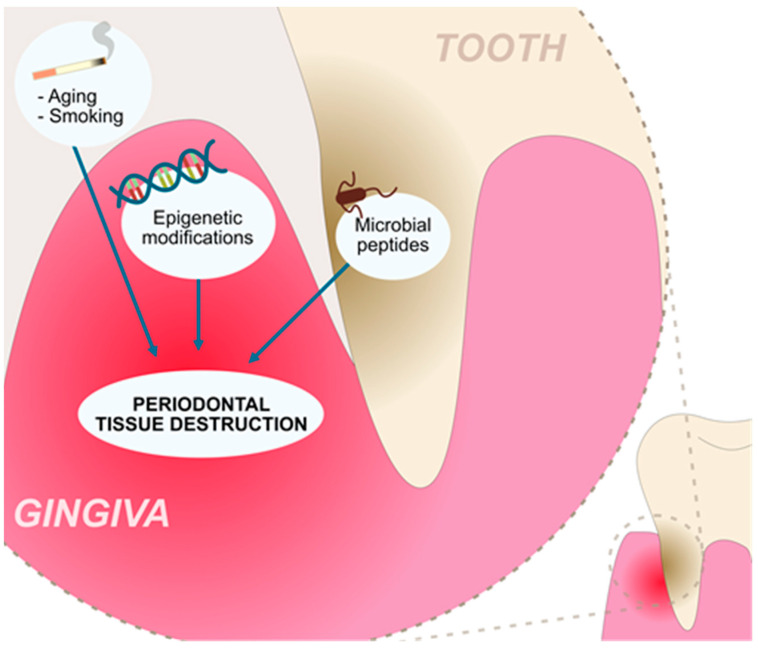
Factors contributing to the chronic inflammation and tissue destruction present in the periodontitis.

Tissues supporting or surrounding the teeth are broadly defined as the periodontium. Diseases of these tissues may develop on the inflammatory, neoplastic, or metabolic backgrounds, among others. Periodontitis (PD) is a prevalent inflammatory disease associated with the activity of the “red complex” bacteria, and it is characterized by a degradation of the alveolar bone and supportive tissues [1,2,3] (Figure 1). Locally, PD can cause tooth loss, but it has also been correlated with the occurrence of oral cancer [4]; thus, investigating the factors that contribute to the development of PD is of great interest. In this review, we try to summarize the gene polymorphisms that are associated with the susceptibility to PD. Furthermore, we discuss the possible epigenetic mechanisms that can modulate the expression of PD-associated genes. Treatment of the disease may include various possibilities, including scaling, root planing, and surgical approaches, among others [5]. Nevertheless, the disease causes loss of periodontal tissues, and potential regenerative therapies are greatly required. Throughout the years, studies have uncovered the important regenerative properties of stem cells; therefore, we aim to present the current evidence on the potentially beneficial roles of stem cells in the treatment of PD.

## 2. Pathogenesis and Genetic Predisposition to Periodontitis

### 2.1. Antimicrobial Peptides

Periodontitis is a state of permanent inflammation in the periodontium. Microbial biofilm and their metabolism cause host cell death and increased turnover, and increased regeneration affects defense responses and epigenetic alterations [6]. Gingival crevicular fluid (GCF) is derived from the periodontal tissue, and its production increases in PD [7]. It consists of many different cells stimulated by interleukin 8, which aimed to produce antimicrobial peptides (AMPs) to control the immune response [6,7]. In PD, the expression of AMPs can be dysregulated [8], and upregulation could suggest an engaged immune response to control pathogen-induced inflammation.

Human β-defensins (HBDs) are AMPs that control innate immune responses. They are produced by gingival epithelial cells and are effective against bacteria, viruses and fungi [9]. They are considered to form pores in the membrane, which can lead to cell death [10]. Additionally, they attract immune cells, such as dendritic cells, and T cells [9]. HBDs are coded by *DEFB* genes, which are located at chromosome 8. In 2019, over 30 *DEFB* genes have been identified [11]. The expression of HBDs can be regulated by various stimuli; for instance, Mathews and colleagues showed that IL-1β and LPS promote HBD-2 expression in gingival keratinocytes [12]. An adenovirus-mediated transfection of the *hBD3* gene stimulated the osteogenic differentiation of the human periodontal ligament cells (PDLCs) and promoted periodontal repair in rat models of periodontitis [13]. Additionally, the administration of HBD3 decreased the expression of matrix metalloproteinase 9 (MMP-9), as well as pro-inflammatory TNF-α and IL-6, in periodontal tissues. Furthermore, HBD3 promotes the expression of IL-10 [14]. Importantly, significantly less colony-forming units of several pathogens, including *Porphyromonas gingivalis*, were observed in the presence of periodontal cells transfected with HBD3 [15].

Single nucleotide polymorphisms (SNPs) are genetic variances of the genome found in a sufficiently large fraction of the population. When an SNP falls within the coding region of the genome, it can change a coded protein’s structure [16]; however, with the degeneracy of genetic code, many SNPs are synonymous, meaning that they do not change the amino acid sequence. Their appearances help to explain the differential susceptibility between individuals regarding an increased risk for different illnesses or for disease resistance [17]. SNPs can alter the way enzymes or receptors work with some profoundly impacting one’s health. Studies have demonstrated that AMP gene polymorphisms can be associated with the susceptibility of PD; for instance, the CC genotype of the rs1800972 *hBD1* polymorphism located in the 5′-UTR region has been found to promote the disease in a Japanese population. Interestingly, reduced HBD1 levels were detected in patients harboring the CC genotype, highlighting the role of genetic variants in protein expression [18]. Importantly, the associations between genetic variants and diseases are highly dependent on the population, which has been depicted in the meta-analysis by Ślebioda et al. The authors found no correlations between genotypes and alleles of rs1800972 with PD [19]. In contrast, the *DEFB4A* rs1339258595 has been found to impact the susceptibility to PD [20]. Moreover, the variations in the copy numbers can predispose an individual to the development of various diseases. For instance, in colonic Crohn disease, the shift toward a reduced number of *HBD-2* copies was noted [21]. In PD, significantly lower *DEFB4* copies were observed in patients with severe disease compared to slight/moderate chronic PD and controls [22]. Furthermore, a large genome-wide association study (GWAS) showed a positive association between *DEFA1A3* rs2738058 SNP and the disease [23].

Lactoferrin is an iron-binding protein effective against a variety of bacteria and is produced in mucus membranes. By binding to iron ions, it inhibits bacterial growth. Pathogens implicated in the pathogenesis of PD have been found to degrade lactoferrin [24]. An early study demonstrated that allele G of the 88A/G polymorphism occurred significantly more frequently in the African American patients with aggressive PD than in controls. Genotypes with allele G showed an elevated risk of disease development (OR = 3.465, *p* = 0.0013). No similar associations were found in the Caucasian population [25]. Moreover, a significant association between the functional polymorphism rs1126478 and aggressive PD has been found in the Taiwanese population [26]. Zupin et al. demonstrated that the GG genotype of the rs1126478 lactoferrin polymorphism was related to chronic PD, while rs1126477 was associated with the development of a less severe condition [27].

Cathelicidin LL-37 is a cationic peptide produced by macrophages and epithelial cells. It neutralizes lipopolysaccharides from Gram-negative bacteria, including *P. gingivalis*, and enhances the migration and activation of neutrophils [28,29]. Elevated concentrations of the cathelicidin LL-37 have been detected in patients with chronic PD [30]. Importantly, smoking seems to decrease the levels of LL-37 in GCF, thus changing the immune responses to pathogens [31]. AMPs are an addition to the epithelial cell barrier to create local defense mechanisms. They have immunomodulatory properties and impact gene expression when the periodontium is exposed to bacterial infection. Studies have shown that AMPs combined show a synergistic effect, significantly increasing their effectiveness during inflammation [8,32].

### 2.2. Interleukins

Interleukins are the group of cytokines involved in the processes of the immune system. Acting through their receptors, these molecules stimulate signaling pathways and mediate inflammation. As their physiological action is crucial for immune responses, any alterations in their amount or action can impair the process and cause persisting inflammation. As a result, inflammatory conditions change the expression profile of periodontal cells [33]. Polymorphisms in interleukin genes can reduce natural defense processes, resulting in developing periodontitis (e.g., damage of periodontal sites, destruction of connective tissue, and loss of alveolar bone). Importantly, due to the abundance of studies examining polymorphisms, multiple meta-analyses have been published.

To begin with, higher levels of IL-1β are detected in patients with PD than in controls [34,35]. Furthermore, its concentrations positively corelate with PD clinical parameters [35]. Multiple studies have evaluated the potential role of polymorphisms in the *IL1* gene. In the meta-analysis by Feng and Liu, which included 12 studies with 1356 patients with chronic PD, significant results were obtained regarding the *IL1A* promoter -889C/T polymorphism. The authors showed that harboring the T allele increased the susceptibility [36]. The TT genotype has previously been found to promote the transcriptional activity of the *IL-1A* gene, which supports the observed results [37]. Similarly, an early study by Shirodaria et al. demonstrated that patients with severe PD harboring the second allele had significantly elevated IL-1α concentrations [38]. Furthermore, allele T was also found to impact susceptibility to peri-implantitis in a Chinese Han but not in a Portuguese population [39,40]; however, the result should be considered with the fact that East Asian and European populations greatly differ in the frequency of the minor allele [41]. A similar study demonstrated that rs17561 (T allele) is associated with PD susceptibility [42].

Similarly, polymorphisms of the *IL-1β* gene have been widely studied in the context of PD. In a meta-analysis including a Chinese population, *IL-1β* C-511T polymorphism was found to influence the susceptibility to PD. Allele T, as well as genotypes TT (vs. CC and vs. CC+CT) and TT+CT (vs. CC) were significantly associated with the disease [43]. Da Silva et al. performed a large meta-analysis of 54 studies investigating the role of rs1143634 in the susceptibility to chronic PD. Allele T was associated with the occurrence of the disease (OR = 1.35, *p* < 0.00001) [44]. Interestingly, in patients with *IL-1B* 3954 C/T polymorphism (genotypes TT, CT), the presence of *Fusobacterium nucleatum* was significantly more common than in patients with the CC genotype. Moreover, the presence of TT genotypes, together with the detection of three species of red complex bacteria, was associated with higher *IL-1β* concentrations [45].

Interleukin-17 (IL-17) is another cytokine investigated in the pathogenesis of PD. IL-17 promotes barrier immunity through mediating tight junction proteins, stimulates the production of AMPs, and takes part in the recruitment of neutrophils. However, an imbalance of IL-17 has been correlated with autoimmune diseases. Dysregulated IL-17 could take part in the pathogenesis of PD as well, as this cytokine can enhance inflammation and promote osteoclast formation [46]. Studies have shown different expression profiles of IL-17 in GCF [47,48]. In an in vivo experiment, the introduction of IL-17 promoted inflammation and bone loss [49], and the reduction in IL-17 levels was observed after treatment of patients with aggressive PD [50]. Moreover, the percentage of mucosal-associated invariant T cells (MAIT) producing IL-17 is higher in PD patients than in controls [51]; thus, the dysregulation of IL-17 could play a role in PD development. Importantly, polymorphisms in the *IL-17* gene have been found to impact susceptibility to the disease; for instance, a meta-analysis by Farmohammadi showed that the AA genotype of the A-197G polymorphism has been associated with an increased odds ratio (OR) [52]. Interestingly, particular genetic profiles may influence the presence of pathogenic bacteria. Linhartova and colleagues demonstrated that allele A of the −197A/G polymorphism has been correlated with the presence of *Tannerella forsythia*, which is one of the “red complex” bacteria [53,54]. However, the novel c. *34 G>A *IL-17F* variant may be implicated in the pathogenesis of PD [55].

### 2.3. Prostaglandin-Endoperoxide Synthase-2 (PTGS2)

Prostaglandin-endoperoxide synthase-2 (PTGS2), also known as cyclooxygenase-2 (COX-2), is an enzyme responsible for creating prostaglandin E2 (PGE2) from arachidonic acid. PGE2 is heavily expressed during inflammation, and its levels are increased in GCF. The PGE2 level can also predict periodontal attachment loss and can be associated with bleeding upon probing [56,57]. Genetic polymorphism in the −765 region has been found to impact the occurrence of PD. Ho and collaborators demonstrated that patients harboring genotypes with allele C had a reduced susceptibility to the aggressive (adjusted OR = 0.071, *p* < 0.0001) and chronic disease (adjusted OR = 0.552, *p* = 0.004) [58]. This polymorphism is functional, and the presence of allele C is associated with reduced promoter activity [59]. In the meta-analysis, the result was not significant in this polymorphism except for in Chinese patients [60]. Interestingly, the *COX-2* polymorphism may influence the expression of other genes. Mesa and colleagues chowed that the rs6681231 polymorphism was correlated with IL-6 positive cells in the connective tissue [61].

### 2.4. Matrix Metalloproteinases

Matrix metalloproteinases (MMPs) are calcium-dependent endopeptidases that degrade extracellular matrix proteins. They also participate in cell metabolism, including proliferation, apoptosis, and the immune response. They are further subdivided into groups, depending on the substrate they operate on [62]. As they are present in all tissues, their role in PD has been assessed many times. Their action is focused on destroying marked substrates; therefore, their overexpression, under-regulation, or gene polymorphisms may be crucial in chronic inflammation, such as PD. An increase in their expression leads to an imbalance between MMPs and their inhibitors, leading to tissue degradation, as higher levels of MMPs have been correlated with PD [63,64].

Specific MMP gene polymorphisms are associated with PD susceptibility. The *MMP1* g.-1607dupG polymorphism (2G/2G and dominant 2G/2G + 2G/1G) was significantly associated with PD [65]. The 2G/2G homozygotes have previously been found to have an elevated expression of MMP-1 [66]; however, according to another meta-analysis, the significant association with -1607 G1/G2 polymorphism was lost except for the recessive model in Asian populations [67].

In another meta-analysis by Weng et al., the authors collected data from 24 case-control studies, and four polymorphisms were included: *MMP*-9-1562 C/T, *MMP*-3-1171 A5/A6, *MMP*-2-753C/T, and *MMP*-8-799 C/T. The *MMP*-9-1562 T allele and TT genotypes were associated with reduced susceptibility to PD. By contrast, *MMP*-3-1171 5A/6A and *MMP*-8-799 C/T were proven to increase the risk. The authors did not find an association between *MMP*-2-753C/T and PD [68]. A study by Bhattari et al. showed that resveratrol, an antioxidant and anti-inflammatory polyphenol, modulates cellular response. Human gingival fibroblasts were stimulated with bacterial LPS to induce the expression of MMPs, and resveratrol suppressed the expression of metalloproteinase in vitro. In addition, the antioxidant agent reduced tissue damage in an in vivo experiment [69].

### 2.5. CD14 Molecules and Toll-like Receptors

Toll-like receptors (TLRs) play a crucial role in the immune system response due to their ability to recognize pathogen-associated molecular patterns (PAMPs) like lipoproteins, flagellin, or lipopolysaccharide (LPS) [70]. This leads to the release of inflammatory cytokines by innate immune myeloid cells [70]. CD14 is known as a pattern recognition receptor (PRR) [71], and it is also considered to be a TLR co-receptor [72]. It can recognize and bind directly with the pathogen’s LPS [73]. Two *CD14* polymorphisms may have an impact on the susceptibility of PD: -159(C/T) and -1359(G/T) [74]. Patients who suffered from moderate periodontal disease less often had the CD14/-1359 G allele than people with advanced illness [75]. Donati et al. showed that patients with periodontal disease were also more often carriers of the -159*C allele in comparison to healthy people [76]. The research focused on the TLR2 and TLR4 polymorphisms found no association with PD development [74].

### 2.6. Human Leukocyte Antigen

The Human Leukocyte Antigen (HLA) genes are located on the major histocompatibility complex (MHC) on the short arm of the sixth chromosome [77,78]. The HLA plays a significant role in the human immune system [78] and is responsible for the presentation of endogenous and exogenous antigens to T lymphocytes [79]. The surface glycoproteins are molecules classified into HLA class I, HLA class II, and class III [74,79], and each of them takes part in a different phase of immune response. Genes that encode the HLA are known to be polymorphic and thus have an influence on the antigen-specific T-cell reaction [80]. Furthermore, they may lead to altered susceptibility to illnesses [79], such as periodontal disease [80]. Studies have focused on the connection between the HLA class I and class II molecules and PD development [74,80]. Mattuella et al. examined the influence of the class I HLA-G gene’s polymorphisms on chronic and aggressive PD, and they found that patients with the chronic disease were more often carriers of the HLA-G 14bp del allele than healthy people [81]. Moreover, another study conducted on a mixed population proves that the polymorphism in HLA class II genes also has an impact on periodontal disease progression; for example, healthy patients were less frequently carriers of at least one of the following alleles in comparison to patients with aggressive PD: DRB1*0401, DRB1*0404, DRB1*0405, or DRB1*0408 [82].

### 2.7. Antibodies

Immunoglobulins (Ig), commonly known as antibodies, are the glycoproteins among which we can distinguish five isotypes: A, D, E, G, and M [83]. Each has a “Y” shape and comprises two heavy and two light chains, which are characterized by variable and constant regions [83,84]. Ig plays a significant role in the human immune system due to its ability to recognize specific antigen molecules by fragment antigen binding (Fab) located on the variable regions [84]. IgG, which includes four sub-classes (IgG1, IgG2, IgG3, and IgG4), occurs most in human serum. Periodontal infection contributes to IgG2 secretion as the primary human immune system reaction to this illness [74]. Saraiva et al. speculated that the increased IgG2 production may inhibit the periodontal infection’s progression. However, their study showed no statistical difference between IgG serum levels and periodontitis exacerbation [85]. Moreover, IgG2 production depends on genetics, specifically on the Gm allele presence, as this allele is responsible for an increased IgG2 secretion. The study by Chung et al. on the Taiwanese population showed that patients with a less frequent Gm allele had an increased risk for chronic periodontitis [86]. Those findings are contradicted by the findings of a similar study conducted on an American population by Hwang et al., where they showed no statistical correlation between the Gm allele and chronic periodontitis risk [87].

### 2.8. Receptor for Antibody Fc Fragment (FcR)

The fragment of the IgG antibody, which is responsible for the immune response’s inception to pathogen recognition, is called the crystallizing fragment (Fc) [88]. The receptor for Ig Fc fragments (FcR) is located on the surface of the hematopoietic cells [89]. Opsonization by IgG pathogens is provoked by FcR bonding with antibodies, the subsequent secretion of inflammatory factors, and the processes of phagocytosis and endocytosis [89]. Among FcR, we can differentiate a few major groups, including FcγRI (CD64), FcγRII (CD32), and FcγRIII (CD16). Kobayashi et al. demonstrated that Japanese patients with inhibitory FcRIIb and stimulatory FcγRIIa genotypes have a greater susceptibility to PD and systemic lupus erythematosus [90]. Furthermore, many polymorphisms in genes encoding the FcγRIla, FcγRIIIa, and FcγRIIIb subgroups may affect patients’ susceptibility to periodontal illnesses [74]. For FcγRIla, two alleles were responsible for expression, R131 and H131, which showed a variable occurrence in patients with AP or CP depending on the examined ethnic population [91]. Another polymorphism involves the NA1 and NA2 alleles for FcγRIIIb [74]. The research conducted on the Japanese population shows that patients who suffer from aggressive PD were more frequently carriers of the N2 allele than healthy patients or patients with CP [92]; however, the association between the presence of N2 and AP occurrence was not observed in any other population examined [93]. FcγRIIIa is encoded by two alleles, V158 and F158 [74]. In this case, the V158 allele was more frequently present in Japanese patients with advanced chronic PD [92], but the research did not prove the association between the AP and V158 or F158 allele occurrence [92].

## 3. Epigenetics

### 3.1. DNA Methylation

As demonstrated in the previous sections, multiple molecules and pathways take part in the pathogenesis of PD. Studies evaluating the genetic aspect of predisposition are difficult to interpret due to their abundance and conflicting results. Importantly, the influence of particular variants may largely depend on the population or sample size. Nevertheless, despite genetic variants, epigenetic mechanisms, such as DNA methylation, histone modifications, or non-coding RNA (ncRNA), are important mediators of gene expression, and these processes could be related to the pathogenesis of PD.

First, the addition of methyl groups occurring in the chromosomal DNA is catalyzed by DNA methyltransferases (DNMTs). Interestingly epigenetic DNA methylation can be conferred by PD-associated pathogens. *P. gingivalis* produces outer membrane vesicles (OMVs), structures that promote apoptosis and the inflammation of periodontal ligament stem cells (PDLSCs), which is an important stem cell subtype with regenerative potential. In a series of experiments, Fan and collaborators demonstrated that *P. gingivalis* OMVs induce this effect through mediating the methylation of p53 in PDLSCs [94]. Studies have shown that patients with PD have an altered methylation profile of various genes compared to healthy controls [95,96]. The status of methylation can greatly impact gene expression. Classically, DNA methylation has been associated with repressed transcription [97]. In line with this statement, the *IL6* promoter was hypomethylated in PD cases compared to controls. Importantly, elevated gene expression of *IL6* was detected in patients with apical PD, and similar findings were observed in the case of *IL1β* [98]. Furthermore, a study by Zhang et al. focused on PTGS2 promoter methylation, comparing periodontally inflamed gingiva vs. non-inflamed gingiva. PTGS2 promoter methylation in periodontitis was found to be increased by five-fold, and its level was inversely associated with PTGS2 transcription [99]. Consequently, the question arises regarding whether DNA methylation-modulating agents could be used in the treatment of PD. Interestingly, the use of decitabine, a DNMT inhibitor, suppressed bone resorption in a PD mice model. The drug was found to suppress osteoclastogenesis, promote osteoblast formation, and stimulate the expression of anti-inflammatory cytokines [100]. Furthermore, evaluating the DNA methylation of specific genes could help in the PD diagnosis process [101]. Polymorphisms of genes encoding enzymes associated with DNA methylation have also been associated with PD. In a study by Asa’ad et al., the authors found that polymorphisms within the *DNMT1, IDH2,* and *TET2* genes were associated with susceptibility to PD [17].

### 3.2. Histone Methylation

Histone modifications represent another important mechanism involved in regulating gene expression. These processes include acetylation, phosphorylation, methylation as well as other modifications. These changes mediate the chromatin structure and binding of effector molecules, which ultimately regulate transcription [102]. In the case of methylation, H3K4me3 is associated with the promotion of transcription. In contrast, H3K9 and H3K27 are thought to repress gene expression. These processes regulate the expression of genes associated with differentiation and inflammation, which are two important mechanisms implicated in the pathogenesis of PD. First, osteogenic induction in the periodontal ligament cells is associated with an initial promotion of H3K4me3 on the *RUNX2* promoter, which is a major marker of osteogenesis differentiation. After seven days of induction, the enrichment decreases and a reduction in the repressive H3K9me3 finishes the osteogenic induction. In the case of alveolar bone precursors, induction is associated with an increasing enrichment of H3K4me3 [103]. Investigating the profiles of histone-methylation markers could be used in the future to modulate methylation to induce osteogenic differentiation and to promote bone regeneration. The application of H3K9 and H3K27 methylation inhibitors significantly promotes osteogenic markers in periodontal ligament fibroblasts [103]. Modulating histone methylation should be explored, considering that pathological conditions present in PD alter epigenetic mechanisms. Promoters of matrix and osteogenic genes are enriched with the pro-transcriptional H3K4me3 in PDLSCs. Stimulation with LPS enhances the presence of repressive H3K27me3 markers [104]. Furthermore, LPS treatment of the periodontal ligament cells promotes the occupancy of H3K4me3 in *IL-6* and IL*-1β* genes [105].

### 3.3. MicroRNA

In recent years, a large number of studies uncovered a major role of ncRNA in gene expression. The dysregulation of these RNA molecules takes part in the pathogenesis of many malignancies and inflammatory diseases. The broad group of ncRNA molecules is subdivided into various categories, including microRNA (miRNA), long non-coding RNA (lncRNA), or circular RNA (circRNA), among others. MiRNAs silence gene expression through binding to the 3′UTR region of targeted mRNA, which stops the translation process. A complex made with mRNA and attached miRNA activates the endonuclease, an enzyme that cleaves phosphodiester bonds in mRNA, making it unusable. Thus, miRNAs can alter cell metabolism and functions depending on mRNA translation.

Since these molecules take part in the pathogenesis of inflammatory diseases, monitoring their expression could be used in the diagnosis process, to evaluate the treatment response, or for future treatment. Importantly, RNA molecules can be derived from fluids and not directly from the inflamed tissue, which creates the opportunity for a liquid biopsy. For instance, a recent study by Alminana-Pastor and collaborators demonstrated the possibility for a miRNA-199b isolated from GCF to become a diagnosis biomarker [106]. Furthermore, Kwon et al. isolated plasma RNA molecules pre- and post-PD treatment, and the authors detected that miR-200c and miR-1304-3p were downregulated in patients compared to controls. Interestingly, the expression of these molecules returned to healthy concentrations after treatment [107]. Thus, these studies suggest that miRNA may help in diagnosis of the disease or in evaluating treatment response. Moreover, the profile of miRNAs can be monitored to detect a bacterial infection. According to Nayar et al., a stimulation of rat gingival tissues with bacterial infection changed the profile of RNA molecule expression [108]. Some studies evaluated the associations between PD-associated pathogens and miRNA profiles. *P. gingivalis* is often described as a leading pathogen in PD and is found in subgingival pockets where it damages periodontal tissue, creating conditions for the development of PD [109,110,111]. It has been shown to strongly stimulate miR-203 and miR-584, resulting in IL-8 upregulation [112,113]. The pathogen induces an up- or downregulation of a number of miRNAs [114,115]. For instance, *P. gingivalis* inhibits miR-205-5p expression and in turn upregulates IL-6 and enhances the JAK/STAT signaling pathway [116]. LPS from *P. gingivalis* also suppresses miR-203 in the periodontal ligament cells. MiR-203 targets activator protein-1, which is a transcription factor involved in the pathogenesis of PD. Therefore, the overexpression of miR-203 induces beneficial effects, such as the promotion of viability, inhibition of apoptosis and reduction in pro-inflammatory mediators [117].

**Figure 2:**
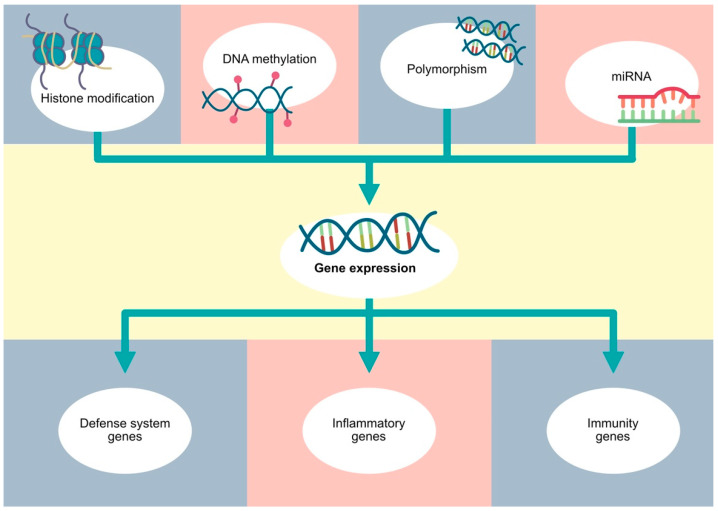
Gene expression depends on various factors, such as polymorphisms, as well as classic epigenetic mechanisms, including DNA methylation, histone modifications and non-coding RNA.

As a Gram-negative bacterium, *P. gingivalis* produces OMVs containing the colonization factors of fimbriae, hemagglutinins, and proteolytic enzymes [118,119]. Those virulence factors have a pro-inflammatory effect on tissue, and notably, they also contain small RNA (sRNA). sRNA was shown to interact with the host immune response to promote bacterial survival. Choi et al. focused their research on three PD-related bacteria, including *A. actinomycetemcomitans*, *P. gingivalis*, and *T. denticola*, and they showed that bacterial OMVs can deliver bacterial sRNA into host cells. They also observed that sRNA produced by *P. gingivalis* altered the host cell’s functions by downregulating interleukin levels and evading the adaptive immune response [120]. Overall, during the pathogenesis of PD, the expression profile of miRNAs changes. Importantly, some of these alterations could be induced by PD-associated pathogens, and understanding these changes could be useful in the diagnosis process. Furthermore, it could be implemented in the treatment strategies, which we will more broadly discuss in the next sections in the context of stem cells. Figure 2 schematically presents various mechanisms associated with the modulated expression of PD-associated genes.

## 4. Stem Cells

### 4.1. Stem Cells and Periodontitis

In recent years, stem cells have become widely investigated as a novel tissue-engineering treatment method. These cells have the ability to proliferate and differentiate into specific cells; therefore, a large number of studies investigated the regenerative properties of these cells [121]. Furthermore, stem cells have immunomodulatory properties which could be applied in the treatment of inflammatory diseases [122]. In addition, these cells can secrete extracellular vesicles (EVs), which are membrane-bound structures that have immunomodulatory properties resembling their parent cells. These vesicles can transfer lipids, peptides, and nucleic acids, which can impact the activity of target cells. Importantly, EVs often transfer ncRNAs, thus contributing to the altered gene expression in recipient cells. The use of stem cell-derived EVs offers a cell-free therapy, which is associated with reduced immunogenicity and the risk of tumorigenesis, as well as a possibility for a long-term storage. Moreover, they can act as drug carriers to precisely deliver the required therapeutics [123].

Mesenchymal stem cells (MSCs) can be isolated from numerous tissues, like bone marrow (BM-MSC), umbilical cord (UC-MSC), or adipose tissue (AD-MSC) [124]. Furthermore, several subtypes of stem cells have been identified from dental tissues. These cells can be derived from dental pulp, periodontal ligaments, exfoliated deciduous teeth and apical papilla [125]. Despite the presence of stem cells in periodontal tissues, their beneficial effects might be suppressed in the pathologic environment of PD. Chronic inflammatory conditions induce pro-inflammatory responses and suppress the osteogenic differentiation of PDLSCs [126]. Cells derived from inflammatory tissues show a reduced expression of OCN and Runx2, which are major genes that take part in osteogenic differentiation [127]. Furthermore, a decreased expression of Runx2 was also found in the osteoblastic lineage cells derived from patients with peri-implantitis [128]. Interestingly, epigenetic modifications seem to play a role in an impaired osteogenic differentiation of PDLSCs derived from inflammatory tissues. Along with the reduced expression of bone development genes, a hypermethylation of these genes was observed [129]. Furthermore, *P. gingivalis* can invade PDLSCs, and the presence of this pathogen reduces the osteogenic differentiation of periodontal ligament cells [130,131]. In addition, *P. gingivalis* stimulates PDLSCs pyroptosis, a form or programmed cell death, to enhance IL-1β secretion and further impair bone homeostasis [132]. Under inflammatory conditions, PDLSCs can further promote inflammation and PD progression [133]; therefore, the application of healthy MSCs or MSCs-secreted EVs could provide beneficial effects on inflammation and tissue regeneration.

### 4.2. The Role of Stem Cells in Periodontal Bone Loss and Tissue Regeneration

During the course of PD, alveolar bone loss is a hallmark of the disease, and regenerative treatment capable of suppressing this process is greatly needed. Alveolar bone loss is a consequence of bone homeostasis imbalance that ultimately promotes osteoclastogenesis. Studies have shown that this process depends on the major inflammatory regulator nuclear factor kappa light-chain enhancer of activated B cells (NF-κB) [134,135] as well as on the presence of the (Nod)-like receptor family pyrin domain-containing 3 (NLRP3) inflammasome [136].

The use of stem cells represents an interesting opportunity to inhibit bone loss and promote tissue regeneration; thus, several studies have investigated their beneficial properties. To begin with, an early study demonstrated that cultured PDLSCs can express markers associated with cementoblasts and osteoblasts. The authors transplanted PDLSCs into surgically generated molar defects in rats and observed that stem cells could attach to the surface of alveolar bone and teeth. In addition, they could integrate into periodontal ligaments, demonstrating that PDLSCs exert regenerative potential [137]. Moreover, the injection of gingiva-derived MSCs (GMSCs) significantly reduced bone loss in mice after four weeks [138]. Similarly, allogeneic BM-MSCs transplantation in rats performed 28 days after ligation-induced PD suppressed the mRNA expression of the receptor activator of NF-κB (RANKL) as well as the population of TRAP-positive cells. Therefore, the authors showed that BM-MSCs treatment could reduce osteoclastogenesis [139]. Interestingly, tissue regeneration can be improved by simulating the natural environment of the extracellular matrix. Balaban et al. investigated the use of GMSCs in fibroin/chitosan hydrogel in PD progression, and the authors observed that this treatment method promoted alveolar bone gain in PD rats [140].

Furthermore, regenerative mechanisms can be induced by paracrine factors derived from MSCs. For instance, cell-free culture medium of PDLCSs and GMSCs demonstrated significant regenerative properties [141,142]. These factors included pro-angiogenic mediators as well as growth factors and cytokines [141]. In addition, the application of EVs derived from MSCs suppressed alveolar bone loss in animal models [127,143,144,145,146]. Recently, a meta-analysis that included 11 preclinical studies investigating the benefits of MSC-EVs demonstrated that the use of these vesicles is associated with a significantly elevated bone volume [147]. The above-mentioned studies showed that stem cells and stem cell-derived EVs impact various pathways to modulate bone homeostasis. For instance, in a study by Lei and colleagues, the authors demonstrated that exosomes derived from healthy PDLSCs could reduce the expression of Wnt pathway members to promote osteogenic differentiation in PDLSCs [127]. These results were confirmed in another study by Nakao et al., in which the authors found that GMSC-derived exosomes could reduce Wnt5 expression via miR-1260b to suppress RANKL expression [146]. Furthermore, MSCs can be modified to induce a secretion of EVs containing an overexpressed molecule. Lai and collaborators prepared BM-MSCs-derived exosomes loaded with miR-26a, and these structures promoted the osteogenic differentiation of BM-MSCs. The injection of exosomal miR-26a into the palatal gingivas of mice with experimental PD significantly enhanced bone regeneration [148]. Interestingly, Ai et al. compared the osteogenic potential of PDLSCs, stem cells derived from dental pulp, as well as dental follicle progenitor cells. The authors detected that osteogenic-related genes were upregulated in PDLSCs and that these cells had the higher osteogenic potential [149]. The co-culture of PDLSCs with dedifferentiated fat cells promoted Runx2 expression in the latter cells, which indicates that PDLSCs can promote the osteogenic differentiation of other cells [150]. Taken together, MSCs and secreted EVs have shown promising results in suppressing alveolar bone loss. Despite the influence on osteoclastogenesis, vesicles derived from healthy cells could enhance the regenerative properties of cells located in the inflamed tissues.

### 4.3. How to Increase the Regenerative Efficacy of Cells in Periodontal Tissues?

In the previous section, we have discussed the beneficial roles of stem cells and EVs in PD-associated bone loss. Nevertheless, periodontal ligament cells with regenerative capabilities seem to be functionally altered under inflammatory and pathogenic conditions. These cells are sensitive to their environment, which translates into altered functionality. For instance, high-glucose and LPS suppress the osteogenic properties of PDLSCs [151]. Moreover, exosomes derived from PDLSCs cultured in high-glucose conditions are less effective at suppressing osteoclastogenesis compared to the structures derived from cells cultured in a normal-glucose environment [143]. Therefore, interventions targeting PDLSCs to improve their regenerative functions, or to prevent inflammatory-induced impairment, might provide benefits in the treatment of PD. These interventions should improve the viability and differencing potential of PDLSCs. Various mechanisms have been investigated, including the use of other stem cells to enhance the regenerative properties of PDLSCs, gene transfection, or the stimulation of PDLSCs with natural or pharmacological agents.

First, PDLSCs can internalize EVs secreted by other cells; for instance, EVs secreted by dental follicle stem cells could promote proliferation and migration as well as osteogenic markers in PDLSCs in an in vitro experiment. Importantly, the application of these vesicles to PD areas in rats promoted new bone formation [152]. Similarly, a co-culture of PDLSCs with EVs derived from human exfoliated deciduous teeth stem cells significantly promoted the expression of osteogenic markers (Runx2, OCN, and Col1) [153]. Second, PDLSCs could be transfected to induce the overexpression of particular genes. For instance, the overexpression of galectin 1 (Gal-1), a β-galactoside-binding protein, rescues LPS-induced depletion of PDLSCs viability [154].

Furthermore, the stimulation of PDLSCs with natural agents could enhance their regenerative properties. In a study by Shi and colleagues, the authors examined the impact of curcumin, an anti-inflammatory and antioxidant product, on the functionality of stem cells. The authors demonstrated that curcumin promoted the viability and osteogenic differentiation of PDLSCs. Interestingly, the authors found that curcumin acted through the transcription factor early growth response 1 (EGR1), and its silencing inhibited the curcumin-induced beneficial effects [155]. Similar results were obtained by Li et al., who demonstrated that betulinic acid promoted the viability and osteogenesis of PDLSCs via EGR1. Transfecting PDLSCs with the EGR1 vector significantly promoted Runx2, OCN, OPN, and Col1 [156]. Therefore, these studies show that EGR1 is an important element in the process of osteogenic differentiation. Previously, a study investigating dental stem cells from apical papilla found that osteogenic differentiation is associated with the upregulation of EGR1. The study suggested that EGR1 mediated the expression of bone morphogenic protein 2 (BMP2) and distal-less homeobox 3 (DLX3) [157]. BMP2 promotes the differentiation of BM-MSCs into osteoblasts, and it has been investigated in bone defects. Interestingly, Tan and collaborators used hydrogel with BMP2, as well as stromal cell derived factor 1 (SDF-1), with chemotactic properties. In this method, SDF-1 promoted the migration of BM-MSCs, while BMP2 stimulated osteogenic differentiation. Importantly, this hydrogel significantly promoted the bone volume in rats with periodontal bone defects [158]. Additionally, PDLSCs have been investigated after the treatment with resveratrol, an anti-inflammatory phytoestrogen, which promoted the viability and osteogenic markers of PDLSCs [159]. Similar results were demonstrated in a recent study by Ma et al. [160]. Importantly, natural agents affect not only PDLSCs gene expression but also their paracrine properties. The stimulation of periodontal stem cells with psoralen, a compound extracted from *Psoralea corylifolia,* changed the pattern of miRNAs secreted in exosomes. Vesicles derived from stimulated cells were associated with a reduced expression of miR-125b-5p, which is a molecule that inhibits osteogenesis [161].

Epigenetic factors also seem to affect PDLSCs function. First, treatment with DNA methyltransferase inhibitor reduced the expression of senescent markers and promoted stemness, which could be associated with a positive modification; thus, the relationship between DNA methylation and MSC functionality in PD should be further explored [162]. Second, an inflammatory environment promotes the expression of lysine demethylase 1A (LSD1), which is an enzyme involved in the histone demethylase process. Under LPS stimulation, silencing LSD1 enhances the expression of ALP, OCN and Runx2. LSD1 was found to reduce the H3K4me2 methylation of osterix transcription factor (OSX) that is involved in osteogenic differentiation. Therefore, LPS promotes the expression of the demethylase enzyme that reduces the pro-transcription methylation marker and ultimately reduces osteogenic potential [163].

In addition, pharmacological agents also regulate the regenerative potential of PDLSCs. For instance, commonly used drugs such as bisphosphonates or acetylsalicylic acid (ASA) also modulate the regenerative potential of stem cells. Di Vito et al. demonstrated that alendronate inhibits PDLSCs’ viability. The authors observed interesting results regarding the expression of osteogenic markers; lower alendronate concentrations suppressed osteogenesis in a shorter time, while a higher concentration (50 μM) strongly promoted OPN expression. Therefore, alendronate might differently regulate PDLSCs’ osteogenesis depending on the stage of differentiation and cellular context [164]. Moreover, zoledronic acid (ZA) was also found to suppress osteogenic markers in PDLSCs [165]. However, stem cells could be modified to reverse the negative effects of bisphosphonates. Duan et al. showed that the upregulation of cAMP response element binding protein 1 (CREB) promotes ZA-inhibited osteogenic differentiating potential [165]. Additionally, the use of ASA with BM-MSCs enhances the alveolar bone repair in rats with experimental PD [166]. Furthermore, the injection of recombinant developmental endothelial locus-1 (DEL-1) protein into the mouse models with ligature-induced PD promoted alveolar bone regeneration. Importantly, in vitro experiments showed that DEL-1 could promote PDLSCs’ osteogenic differentiation under inflammatory and high-glucose conditions [151].

Importantly, the pathogenesis of PD is also associated with oxidative damage. The generation of reactive oxygen species (ROS) is correlated with both physiological and pathological properties, as these molecules can kill microorganisms as well as host cells [167]. Scavenging ROS using antioxidant agents or platforms could enhance the osteogenic features of PDLSCs [168,169]. For instance, the overexpression of superoxide dismutase 2 in PDLSCs stimulated the expression of osteogenic markers. Importantly, the use of transfected cells was associated with a greater bone volume in rats with alveolar bone defects [169].

Taken together, the above-mentioned studies demonstrated that various methods could be used to improve the regenerative properties of PDLSCs. Hypothetically, PDLSCs could be collected, transfected, or treated with pharmacological or natural agents and then re-introduced to periodontal defect areas. Moreover, interventions targeting the pathologic environment in periodontal tissues can also be introduced to elevate the regenerative properties, such as ROS scavenging. Further studies are required to investigate the impact of frequently administered drugs on the regenerative potential of PDLSCs and what the possible mechanisms to reverse the negative impacts of these agents on osteogenic differentiation are.

### 4.4. The Role of Stem Cells in Inflammation

The pathogenesis of PD is associated with dysbiosis and inadequate immune reactions, leading to chronic inflammation [170]. Inflammatory processes are closely linked with pathological mechanisms in PD, such as an increased presence of pro-inflammatory macrophage subtypes [171]. An inflammatory environment negatively impacts the functionality of stem cells and promotes bone loss. The application of stem cells or their paracrine factors exert beneficial immunomodulatory properties, which could enhance treatment and tissue regeneration in PD.

An important and potentially beneficial strategy would be to modulate macrophage polarization. Few studies examined the role of macrophage polarization on the osteogenic differentiation of stem cells. To begin with, the conditioned medium of macrophages stimulated with LPS suppresses the osteogenic potential of PDLSCs by reducing osteogenic markers, such as ALP and OCN [172]. Another study showed that exosomes derived from two macrophage phenotypes differently mediate the osteogenic potential of PDLSCs. M1-derived structures reduced ALP and OCN, while M2 macrophages released EVs that promoted the expression of those markers [173]. Moreover, Kang et al. showed that EVs derived from macrophages can differently impact the osteogenic properties of MSCs. Vesicles derived from the M1 pro-inflammatory phenotype seem to negatively impact the BMP2 signaling pathway in contrast to structures secreted by M0 and M2 subtypes. These observations result from an altered pattern of miRNA expression in secreted vesicles. EVs derived from M1 macrophages contained miR-155, while miR-378a molecules were abundant in the vesicles secreted by the M2 subtype [174]. The expression of miR-155 has been found to be upregulated in the saliva of PD patients and is positively correlated with PD severity [175]. In line with the results of the former study, the administration of miR-378a-3p mimics promoted the osteogenic differentiation of BM-MSCs derived from aged rats [176]. Furthermore, M2 macrophages promoted the cementoblastic differentiation of PDLSCs [177].

Therefore, promoting M2 anti-inflammatory polarization represents a potential treatment strategy, and researchers have been investigating the role of stem cells or their paracrine factors in this process. First, the conditioned medium of PDLSCs promoted M2 macrophage polarization and the production of anti-inflammatory cytokines [178,179]. Moreover, dental pulp stem cells, GMSCs, AD-MSCs, or EVs derived from stem cells have been also found to promote M2 macrophage polarization [144,146,180]. Interestingly, macrophage polarization is associated with ROS activity. AD-MSCs could modulate macrophages through the regulation of ROS and antioxidant molecules. A co-culture of AD-MSCs with macrophages promoted the expression of antioxidant nuclear factor (erythroid-derived 2)-like 2 (NRF2), which is a major regulator of cellular antioxidant properties [172]. BM-MSCs-derived exosomes suppress inflammatory responses in LPS-stimulated macrophages, and these results could be attributed to the abundant miRNA molecules present in EVs. For instance, BM-MSCs-exosomes transfer miR-451a, which is a molecule that has been previously found to induce anti-inflammatory responses [145,181,182,183]. Nevertheless, the role of RNA molecules might depend on the cellular context, as the same molecules can induce various pathways. Interestingly, beneficial effects may be achieved through the use of apoptotic EVs (apoEVs), which are vesicles that are secreted during the process of apoptosis. BM-MSCs-derived apoEVs could reduce the inflammatory responses of *P. gingivalis* LPS-stimulated macrophages via suppressing the NF-κB pathway [184]. Furthermore, dental pulp stem cell-derived exosomes with chitosan hydrogel suppress inflammation in mice with experimental PD by inhibiting the intensity of the Western blot signal of NF-κB p65 and MAPK p38 molecules. The secreted vesicles also promote the anti-inflammatory polarization of macrophages via the transfer of miR-1246 [185]. Interestingly, this RNA molecule also takes part in T cell homeostasis. Importantly, impaired balance between the Th17 cells, regulatory T cells (Tregs) and their cytokines is observed in PD [186,187]. The stimulation of BM-MSCs with *P. gingivalis* LPS reduces the expression of miR-1246 in BM-MSCs-EVs. The overexpression of miR-1246 suppressed the Th17 population and promoted Treg cells [187]. As previously mentioned, PDLSCs act differently upon inflammatory conditions. Healthy cells seem to negatively impact the percentage of Th17 cells. In contrast, a co-culture of peripheral blood mononuclear cells with PDLSCs derived from inflamed periodontal ligaments increases the Th17 cells population [188]. Another study demonstrated that stem cell-derived EVs also influence the presence of Th17 cells. A treatment of PDLSCs with LPS reduced the expression of miR-205-5p in stem cells and in their exosomes. Exosomes overexpressing miR-205-5p suppressed pro-inflammatory mediators (TNF-α, IL-1β, IL-6) in LPS-stimulated rats. Moreover, miR-205-5p in exosomes reduced the percentage of Th17 cells and promoted the population of Treg [189]. Importantly, PD treatment could be enhanced by suppressing the inflammatory responses of PDLSCs. Recently, Wu et al. showed that bomidin, a recombinant AMP, could reduce inflammatory cytokines in murine PD models. The authors demonstrated that it could promote TNF-α-diminished PDLSCs proliferation and migration. Furthermore, it could reduce the expression of pro-inflammatory mediators. The investigated AMP suppressed PDLSCs ferroptosis, which is a type of programmed cell death [190]. Taken together, stimulating the anti-inflammatory cellular profile might induce various beneficial effects, including changing the pattern of EVs cargo that could potentially promote osteogenic stem cell differentiation. Table 1 summarizes the beneficial effects of stem cells and EVs in alveolar bone loss and inflammation.

### 4.5. Clinical Studies

In the previous sections, we have discussed the various roles of stem cells that could promote tissue regeneration and suppress inflammation. As demonstrated in multiple studies, stem cells and EVs showed promising results in preclinical studies. Importantly, MSCs have been also investigated in clinical settings. First, the promising efficacy of stem cells has been demonstrated in case reports describing intrabony defects [191,192]. Second, the most recent meta-analysis performed by Nguyen-Thi et al. included a total of 15 clinical trials investigating the efficacy of stem cells in periodontal tissue regeneration. The analyses showed that stem cell therapy reduced clinical attachment level and probing depth together with radiographic intrabony defect depth. Furthermore, the use of stem cells promoted mineralized bone formation [193]. Therefore, current evidence demonstrated the clinical efficacy of stem cells. Nevertheless, a cell-free therapy is another exciting concept which could eliminate the disadvantages of cellular treatments.

## 5. Conclusions

PD is a chronic inflammatory disease with a complex pathogenesis. In this review, we have tried to demonstrate the important impact of genetics and mechanisms modulating gene expression on PD susceptibility and progression. Examining genetic polymorphisms may select particular populations with an increased risk of developing PD. Moreover, the further understanding about epigenetics may find use in the diagnosis or treatment of PD.

Tissue degeneration is an important consequence of PD, and regenerative therapies are greatly needed. Multiple studies have demonstrated that the use of stem cells represents an interesting and promising method to regenerate damaged tissues and suppress chronic inflammation. Stem cells have already started to be investigated in clinical settings, and these studies show the beneficial effects of stem cell therapy. Additionally, paracrine factors secreted by stem cells represent an interesting cell-free therapy which could bypass the potential disadvantages of a cell-based treatment. Furthermore, PDLSCs, which are present in the periodontal tissues, are functionally impaired under pathological conditions. Their regenerative potential could be enhanced by gene transfection or stimulation with natural compounds or pharmacological agents. Genetics and epigenetics are associated with the susceptibility and pathogenesis of PD. Nevertheless, more studies should evaluate how stem cells modify gene expression in periodontal cells and whether carriers of particular genetic polymorphisms differently respond to cellular-based regenerative treatment.

## Figures and Tables

**Table 1:** Summary of mechanisms associated with stem cells and alveolar bone loss and inflammation in periodontitis.

Mechanism	Therapeutic	Mechanism	References
Alveolar bone loss	PDLSCs	PDLSCs injected into surgically generated molar defects integrated into periodontal ligament and could attach to the surface of teeth and alveolar bone.	[137]
	GMSCs	Injection of GMSCs could suppress alveolar bone loss.	[138]
	BM-MSCs	Allogeneic BM-MSCs transplantation reduced the expression of RANKL and the TRAP-positive cell population.	[139]
	GMSC with hydrogel	Local administration of GMSCs in fibroin/chitosan hydrogel promoted alveolar bone gain.	[140]
	PDLSC culture medium	Transplantation of PDLSC culture medium promoted periodontal tissue regeneration.	[141]
	GMSC and PDLSC culture medium	Application of GMSC and PDLSC promoted new bone formation.	[142]
	Extracellular vesicles	Exosomes derived from PDLSCs promoted the regeneration of alveolar defects by stimulating osteogenic differentiation.	[127]
	Extracellular vesicles	Exosomes derived from PDLSCs suppressed alveolar bone loss by suppressing osteoclast formation in mice.	[143]
	Extracellular vesicles	Exosomes derived from dental pulp stem cells suppressed alveolar bone loss.	[144]
	Extracellular vesicles	The application of exosomes derived from BM-MSCs was associated with reduced bone loss compared to the saline group.	[145]
	Extracellular vesicles	GMSC-derived exosomes reduced alveolar bone loss in PD models.	[146]
	Extracellular vesicles	BM-MSCs-derived exosomes loaded with miR-26a promoted osteogenic differentiation of BM-MSCs and enhanced bone regeneration in vivo.	[148]
Inflammation	Stem cells and their paracrine products	Stem cells and their paracrine products promote anti-inflammatory macrophage phenotype.	[144,146,172,178,179]
	Extracellular vesicles	BM-MSCs secreted exosomes inhibited inflammatory responses in macrophages stimulated with *P. gingivalis.*	[145]
	Apoptotic extracellular vesicles	Apoptotic extracellular vesicles suppress inflammatory responses in *P. gingivalis* LPS-stimulated macrophages.	[184]
	Extracellular vesicles	Dental pulp stem cell-derived exosomes with chitosan hydrogel suppress inflammation in PD mice and promote the anti-inflammatory macrophage phenotype.	[185]
	Extracellular vesicles	Stem cells-derived EVs overexpressing miR-1246 can enhance Th17/Treg homeostasis.	[187]
	Extracellular vesicles	Exosomes containing miR-205-5p inhibit the expression of pro-inflammatory cytokines in LPS-stimulated rats and reduce the population of Th17 cells.	[189]

## Data Availability

Not applicable.
